# Salvigenin, a Trimethoxylated Flavone from *Achillea Wilhelmsii* C. Koch, Exerts Combined Lipid-Lowering and Mitochondrial Stimulatory Effects

**DOI:** 10.3390/antiox10071042

**Published:** 2021-06-29

**Authors:** Elena Serino, Azam Chahardoli, Nadia Badolati, Carmina Sirignano, Fereshteh Jalilian, Mahdi Mojarrab, Zahra Farhangi, Daniela Rigano, Mariano Stornaiuolo, Yalda Shokoohinia, Orazio Taglialatela-Scafati

**Affiliations:** 1 Department of Pharmacy, School of Medicine and Surgery, University of Naples Federico II, Via Montesano 49, 80131 Naples, Italy; e.serino96@gmail.com (E.S.); nadia.badolati@unina.it (N.B.); carmina.sirignano@unina.it (C.S.); drigano@unina.it (D.R.); 2 Department of Biology, Faculty of Science, Razi University, Kermanshah 6714414971, Iran; a.chahardoly@gmail.com; 3 Pharmaceutical Sciences Research Center, Kermanshah University of Medical Sciences, Kermanshah 6715847141, Iran; fe.jalilian@gmail.com (F.J.); mahdi.mojarab@gmail.com (M.M.); 4 Student Research Committee Pharmaceutical Sciences Research Center, Kermanshah University of Medical Sciences, Kermanshah 6715847141, Iran; farhangi_zr@yahoo.com; 5 Ric Scalzo Institute for Botanical Research, Southwest College of Naturopathic Medicine, Tempe, AZ 85282, USA

**Keywords:** *Achillea wilhelmsii*, phytochemical analysis, sesquiterpenoids, metabolic syndrome, mitochondrial stimulatory activity

## Abstract

Phytochemical analysis of the Iranian plant *Achillea wilhelmsii* led to the isolation of 17 pure secondary metabolites belonging to the classes of sesquiterpenoids and phenolics. Two of these compounds, named wilhemsin (**7**) and wilhelmsolide (**9**), are new sesquiterpenoids, and the first shows undescribed structural features. Their structures were elucidated through extensive spectroscopic analysis, mainly based on 1D and 2D NMR, and chemical derivatization. Starting from plant traditional use and previous reports on the activity of the plant extracts, all the pure compounds were evaluated on endpoints related to the treatment of metabolic syndrome. The sesquiterpene hanphyllin (**8**) showed a selective cholesterol-lowering activity (−12.7% at 30 µM), santoflavone (**13**) stimulated glucose uptake via the GLUT transporter (+16.2% at 30 µM), while the trimethoxylated flavone salvigenin (**14**) showed a dual activity in decreasing lipid levels (−22.5% palmitic acid biosynthesis at 30 µM) and stimulating mitochondrial functionality (+15.4% at 30 µM). This study further confirms that, in addition to the antioxidants vitexin, isovitexin, and isoschaftoside, *A. wilhelmsii* extracts contain molecules that can act at different levels on the metabolic syndrome symptoms.

## 1. Introduction

Metabolic syndrome is a complex cluster of disorders including dyslipidemia, diabetes mellitus, obesity, osteoporosis, and cardiovascular diseases affecting one-quarter of the world’s adult population. Compared to the healthy population, patients present a higher risk to die from heart disease or stroke [1]. Chronic inflammation, insulin resistance, production of abnormal adipocytokines such as tumor necrosis factor α, interleukin-1 (IL-1), IL-6, leptin, and adiponectin are all markers of metabolic syndrome at the cellular level [2].

Lifestyle modification remains the initial intervention of choice for such conditions, but pharmacological treatment is considered for those patients whose risk factors are not adequately reduced with lifestyle changes. Although there is no shortage of pharmacotherapies for the prevention and treatment of metabolic disorders, in several cases the severe adverse effects, their high cost, and insufficient accessibility hamper general use. An increasing number of studies have indicated that some natural products are able to ameliorate metabolic syndrome and its risk factors [3], supporting their eligibility as alternative strategies for treatment. Rigorous research to determine effective plant-derived metabolites, and their cellular mechanisms is needed to design animal and clinical trials.

*Achillea wilhelmsii* C. Koch is an herbaceous plant belonging to the Asteraceae family (tribe Anthemidae), widely distributed in Asia, especially in Iran (where it is named “Berenjasf”), Pakistan, and the Indian peninsula. This plant holds a remarkable place in traditional Persian medicine, with a particular indication for the treatment of pulmonary affections, and it is used by the local population for a variety of ailments [4]. Recent investigations have substantiated these traditional uses with a scientific approach and activity on irritable bowel syndrome and ulcerative colitis [5,6], and improvement of oxidative stress-related myocardial injury [7] have been demonstrated. Asgary et al. [8] have reported a significant activity of *A. wilhelmsii* extracts in inducing a decrease in triglycerides and LDL-cholesterol levels after 2–4 months of the administration, a result supported by Khazneh et al. [9] in a more recent investigation. However, both these promising studies were carried out mainly on plant extracts and failed to identify an unambiguous correlation between the extract activity and a specific metabolite or a class of compounds. 

Herein, we report the results of a comprehensive phytochemical investigation of *A. wilhelmsii* aerial part that led to the identification of 17 secondary metabolites (**1**–**17**), including the not yet described sequiterpenoids wilhelmsin (**7**) and wilhelmsolide (**9**). The structural elucidation for these new secondary metabolites, including peculiar structural features, was as well performed. All the isolated compounds have been evaluated in pure form for their lipid-lowering activity, glucose uptake modulating activity, and mitochondrial stimulatory activity unveiling an interesting dual activity for the trimethoxylated flavone salvigenin (**14**).

## 2. Materials and Methods

### 2.1. General Procedures

Optical rotations (CHCl_3_ and MeOH) were measured at 589 nm on a JASCO P2000 polarimeter. ^1^H (400 and 600 MHz) and ^13^C (100 and 150 MHz) NMR spectra were measured on a Bruker spectrometer. Chemical shifts are referenced to the residual solvent signal (CDCl_3_: δ_H_ 7.26, δ_C_ 77.0; CD_3_OD: δ_H_ 3.31, δ_C_ 49.3). Homonuclear ^1^H connectivities were determined by COSY experiments; one-bond heteronuclear ^1^H-^13^C connectivities by the HSQC experiment; two- and three-bond ^1^H-^13^C connectivities by gradient-HMBC experiments optimized for a ^2,3^*J* value of 8 Hz. Through-space ^1^H connectivities were obtained using a ROESY experiment with a mixing time of 500 ms. Low- and high-resolution ESIMS were performed on a LTQ OrbitrapXL (Thermo Scientific, Walthman, MA, USA) mass spectrometer. Medium-pressure liquid chromatography was performed on a Büchi (Switzerland) apparatus using a silica gel (7-230 mesh) column; separations were monitored by TLC on Merck 60 F254 (0.25 mm) plates and were visualized by UV inspection and/or staining with 5% H_2_SO in ethanol and heating. HPLC was conducted on a Young Lin apparatus equipped with PDA detector (YL 9160) using Vertisep (Reversed-phase, RP18 250 by 30 mm) and Eurospher II (Normal phase, Si 250 by 20 mm) columns with flow rate of 10 mL/min and on a Knauer apparatus equipped with a refractive index detector and Luna (normal SI60 and RP-18 phases, 250 by 4 mm) (Phenomenex, Torrance, CA, USA) columns with 0.7 mL/min as flow rate.

### 2.2. Plant Collection, Extraction, and Purification

Aerial parts of *Achillea wilhelmsii* C. Koch were collected from the mountain of Terazag Abdollah village, Islamabad-e Gharb city, Kermanshah province, Iran in spring 2017. The plant species was identified by Dr. Nastaran Jalilian, Agricultural and Natural Resources and Education Center, Kermanshah, Iran. A voucher specimen of the plant was deposited at the herbarium of the Department of Pharmacognosy, School of Pharmacy, Kermanshah University of Medical Sciences, Kermanshah, Iran (No. 2729). 

The plant samples were dried at ambient temperature and crushed and powdered using an electrical mill. The air-dried aerial parts of *A. wilhelmsii* (565 g) were extracted with 350 mL of *n*-hexane, dichloromethane, acetone, and methanol, respectively, by a Soxhlet apparatus for 4 h each run. After every extraction, the plant material was dried again and extracted with the next solvent. Thus, we obtained dichloromethane (19.3 g), acetone (8.3 g), and methanol (55.7 g) dried extracts that were stored at −20 °C, until further analysis.

A portion of the dichloromethane extract (5.0 g) was subjected to silica gel vacuum liquid chromatography (VLC) using as gradient mobile phase chloroform:MeOH: H_2_O (29:1:0.1) to achieve 4 fractions (DCM1-4). DCM2 (2.15 g) was further fractioned with silica column using as gradient mobile phase *n*-hexane:EtOAc from 8:2 to 1:9, obtaining 9 fractions (DCM2-A to DCM2-I). DCM2-D was further purified using a semi-preparative HPLC (gradient from 70% MeOH in water to 100%) to achieve pure leukodin (**1**, 62.0 mg). DCM2-F was purified in the same conditions as DCM2-D and afforded pure hanphyllin (**8**, 23.2 mg), penduletin (**11**, 43.0 mg), artemetin (**12**, 32.6 mg), and salvigenin (**14**, 88.5 mg). DCM2-G was purified in the same conditions as DCM2-D and afforded pure santoflavone (**13**, 11.6 mg). 

The acetone extract was fractionated by reversed phase VLC with a gradient of MeOH:H_2_O (1:9 to 100%) to obtain twelve fractions (ACET1 to ACET12). The fraction ACET1-2 were combined and further analyzed by RP18 column eluted by methanol in water (10:90 to 100:0), which obtained 7 fractions of ACET1A-G. Fractions ACET1A-C (0.22 g) and ACET1A-D (0.17 g) were combined and purified with semi-preparative HPLC (MeOH 30 to 40% in H_2_O) to obtain pure chlorogenic acid (**10**, 29.1 mg); fraction ACET1E (0.19 g) was purified in the same conditions to afford artecanin (**3**, 8.2 mg), artecaninhydrate (**4**, 7.1 mg), artemargyinolide B (**5**, 9.1 mg), and chloroklotzchin (**6**, 4.1 mg). Fraction ACET1F (0.12 g) was also purified in the same conditions to obtain a crude fraction that was chromatographed by RP-18 HPLC (eluent MeOH-H_2_O 4:6) to afford the new wilhelmsin (**7**, 6.9 mg).

The methanol extract was separated into 12 fractions (MET1 to MET12) using VLC method with a gradient of MeOH:H_2_O (5:95 to 100:0) as a mobile phase. From these samples, MET7 (1.9 g), was purified by semi-preparative HPLC (40 to 50% MeOH in water), to obtain deacetylmatricarin-8-*O*-*β*-glucopyranoside (**2**, 8.3 mg), vitexin (**15**, 11.4 mg), isovitexin (**16**, 9.4 mg), isoschaftoside (**17**, 7.7 mg), and the new wilhelmsolide (**9**, 4.4 mg). 

*Wilhelmsin* (**7**). Colorless amorphous solid, [α]_D_ + 4.2 (c 0.06, MeOH); ^1^H NMR (CD_3_OD, 600 MHz): see Table 1; ^13^C NMR (CD_3_OD, 150 MHz): see Table 1; ESIMS *m/z* 386 [M + Na]^+^; HRESIMS *m/z* found 386.1573 (C_19_H_25_NO_6_Na requires 386.1580).

*Wilhelmsolide* (**9**). Colorless amorphous solid, [α]_D_ + 24.7 (c 0.05, CDCl_3_); ^1^H NMR (CDCl_3_, 400 MHz): see Table 2; ^13^C NMR (CDCl_3_, 100 MHz): see Table 2; ESIMS *m/z* 345 [M + Na]^+^; HRESIMS *m/z* found 345.1313 (C_17_H_22_O_6_Na requires 345.1314).

### 2.3. Mosher Ester Derivatization of Wilhelmsolide

Two aliquots of weilhelmsolide (**9**) (1.0 mg) were treated with *R*-MTPA and *S*-MTPA chloride (30 μL) in 400 μL of dry pyridine with a catalytic amount of DMAP overnight at rt. Then, the solvent was removed, and the products were purified by HPLC (*n*-hexane–EtOAc, 95:5) to obtain, respectively, the *S*-MTPA ester (**9a)** (0.9 mg) and the *R*-MTPA ester (**9b**) (1.1 mg).

### 2.4. Reagents for Biological Assays

Chemicals and reagents used for metabolite extraction were all HPLC grade. Water was treated in a Milli-Q water purification system (Millipore, Bedford, MA, USA) before use. The standards used for the identification of intracellular metabolites were from Sigma Aldrich (Taufkirchen, Munich, Germany). MitoTracker Red CMXRos (M7512, Invitrogen, Carlsbad, CA, USA) used for staining of mitochondria was reconstituted in DMSO and 1mM stock aliquots were stored at −20 °C before use. PBS (A0965-9010), CaCl_2_ (A3779-1000), TritonX-100 (A1388-0500) were all from Applichem (Darmstadt. Germany). Insulin (I6634), glycine (50046), methanol (322415), (2-(*N*-(7-Nitrobenz-2-oxa-1,3-diazol-4-yl)amino)-2-Deoxyglucose)(2-NBDG, 72987), 2-Deoxyglucose (2-DG;D6134), and (4′,6-diamidino-2-phenylindole) (DAPI, D8417) were from Sigma Aldrich (Taufkirchen, Germany). Formaldehyde (7040) was from JTBaker (Deventer, The Netherlands). Apple polyphenolic extract was produced as described in [10]. Insulin (12585014) was from Thermo Fisher Scientific.

### 2.5. Cell Culture and D_2_O Labeling 

HuH7, human hepatoma cells 7 clone 5 (passage 49), were obtained from Ceinge Biotecnologie Avanzate (Naples, Italy). These cells possess a stable hepatic phenotype and can be kept in culture for a long time without accumulating epigenetic changes or losing their differentiated state and function (production of plasma proteins). Cells were cultured (till passage 80) in Dulbecco modified Eagle medium (DMEM) (41965-039, GIBCO, Thermo Fisher Scientific, Waltham, MA, USA) supplemented with 10% FBS (10270, GIBCO), glutamine (35050-061, GIBCO), penicillin, and streptomycin (15070-063, GIBCO) in a cell culture incubator at 37 °C and with 5% CO_2_. When indicated HuH7 cells were stained with the neutral lipid stain BODIPY™ 493/503 (4,4-Difluoro-1,3,5,7,8-Pentamethyl-4-Bora-3a,4a-Diaza-s-Indacene) (Thermo Fisher Scientific). Briefly, cells were fixed in 3.7% formaldehyde diluted in PBS for 30 min, to be then permeabilized with 0.1% Tryton X100, stained with 2 μM BODIPY 493/503 and visualized under a fluorescence microscope For D_2_O labeling 2 × 10^6^ HuH7 cells were cultivated in a medium supplemented with 5% D_2_O (Sigma Aldrich St. Louis, MO, USA). When indicated, tested compounds (30 μM), Simvastatin (12 μM) or vehicle (DMSO) were added to the cultures for 72 h. 

### 2.6. GC-MS for Biological Tests

For GC-MS analyses, 2 × 10^6^ HuH7 cells were scraped in 400 μL ice-cold water to be then supplemented with 400 μL methanol and 500 μL chloroform. After vigorous vortexing, samples were centrifuged at 10,000× *g* for 10 min at 4 °C. Organic phases were collected and dried. Sample was solubilized in pyridine (50 μL) and derivatized with 25 μL of N,O-Bis(trimethylsilyl(TMS)trifluoroacetamide with trimethylchlorosilane (BSTFA+TMS) with a reaction time of 90 min. GC-MS analyses were carried out on a Shimadzu GCMS 2010plus (Kyoto, Japan) with the following parameters. Injection temperature 280 °C, Ramp 0–1.00 min 100 °C, 1.00–6.00 min 100–320 °C, hold for 2.33 min, column flow 1.10 mL/min, linear velocity 39 cm/s. Helium gas was used. Ion source temperature 200 °C, Interface 320 °C, Solvent cut 5.9 min, Scan 35–600 *m*/*z*. Detector voltage 0.1 kV. Separation was performed on an Agilent (Santa Clara, CA, USA) SIL-8, 30 m by 0.25 mm, 0.25 μm. Then, 1 μL was injected, split ratio 1:10.

### 2.7. Mitochondrial Activity

Mitochondria activity of HuH7 cells was measured upon incubation with the Mitochondrial probe, MitoTracker^®^ Red CMXRos (Thermo Fisher Scientific). A dye working solution was prepared by diluting the probe in DMEM to yield a final concentration of 100 nM. For staining of in vitro samples, HuH7 were rinsed twice in PBS before adding the dye. Cells were incubated in the presence of the probe for 45 min in a cell incubator at 37 °C and 5% CO_2_. At the end of the incubation, cells were rinsed three times in DMEM and once in PBS, fixed in 3.7% formaldehyde for 30 min to be then permeabilized in 0.1% Triton X-100 in PBS and stained with the nuclear dye DAPI. Mitochondrial fluorescence was measured in a Perkin Elmer Envision 2105 Multiplate reader (Perkin Elmer), using the inbuilt monochromator and the following parameters: λ excitation 579 nm, λ emission 599 nm for MitoTracker, λ excitation 351 nm, and λ emission 450 nm for DAPI correlated with the total number of cells in each well and was used for normalization.

### 2.8. 2-NBDG Glucose Uptake Assay on HuH7 Cells

HuH7 cells were plated (5 × 10^3^/well) in a black, clear bottom, 96-well microtiter plate (Perkin Elmer, Waltham, USA) in a final volume of 100 μL/well of culture medium. Once cells had reached 80–90% of confluence, the culture medium was carefully removed and replaced with 100 μL of HBSS containing 100 μM 2-DG, 0.4 g/L BSA, and 1.3 mM CaCl_2_ (in the absence of any growth factors or FBS) and when indicated the tested compounds. Plates were incubated at 37 °C for 1 h. Cell medium was replaced with the same HBSS supplemented with 6 μM 2-NBDG. Plates were incubated with the fluorescent probe for 45 min to be then washed twice in PBS. Uptake of 2-NDBG was measured in a Perkin Elmer Envision 2105 Multiplate reader (Perkin Elmer), using the inbuilt monochromator and the following parameters: λ excitation 471 nm, λ emission 529 nm, and monochromator cut off 360 nm. After the measurement of 2-NDBG, cells were fixed in 3.7% paraformaldehyde for 30 min to be then permeabilized in 0.1% Triton X-100 in PBS and stained with the nuclear dye DAPI (30 μM). This second fluorescence measurement correlates with the total number of cells in each well and was used for normalization. DAPI fluorescence was measured using the following parameters: λ excitation 351 nm and λ emission 450 nm. Data analysis for glucose uptake is reported as the ratio between intracellular 2-NDBG fluorescence and DAPI fluorescence ± s.d.

### 2.9. Statistical Analysis

Rate of deuterium incorporation in newly synthesized cholesterol molecules was expressed as percentage of difference in cholesterol biosynthesis compared to untreated samples; the rate of deuterium incorporation in newly synthesized palmitic acid molecule expressed as percentage of difference in palmitic acid biosynthesis compared to untreated samples; variation in mitochondrial difference in potential expressed as percentage of increase in mitochondrial activity compared to untreated samples; increase in glucose uptake via GLUT transporters expressed as percentage of glucose uptake compared to untreated samples. Comparisons and differences were analyzed for statistical significance by one-way ANOVA and Bonferroni multiple comparisons test with a single pooled variance. Statistical analysis was performed using GraphPad Prism (GraphPad Software 7.03, San Diego, USA). Values were considered statistically different when *p* value were * < 0.01, ** < 0.05, *** < 0.001; and considered not statistically different when *p* value was > 0.05.

## 3. Results

### 3.1. Chemical Analysis and Structure Elucidation of Wilhelmsin and Wilhelmsolide

Aerial parts of *A. wilhelmsii* were defatted with *n*-hexane and subjected to consecutive Soxhlet extraction with dichloromethane (DCM), acetone, and methanol (MeOH) to obtain an exhaustive extraction of secondary metabolites. A previous report from our group had estimated the highest total phenolic content (gallic acid equivalent, GAE) and total flavonoid content (quercetin equivalent, QE) of *A. wilhelmsii* extracts to be 1393 μg GAE and 707 μg QE at concentration 300 µg/mL, respectively [11]. Thus, as expected, DCM and acetone extracts proved to be rich in apolar terpenoids and flavonoids, while the MeOH extract concentrated the glycosylated compounds.

**Figure 1:**
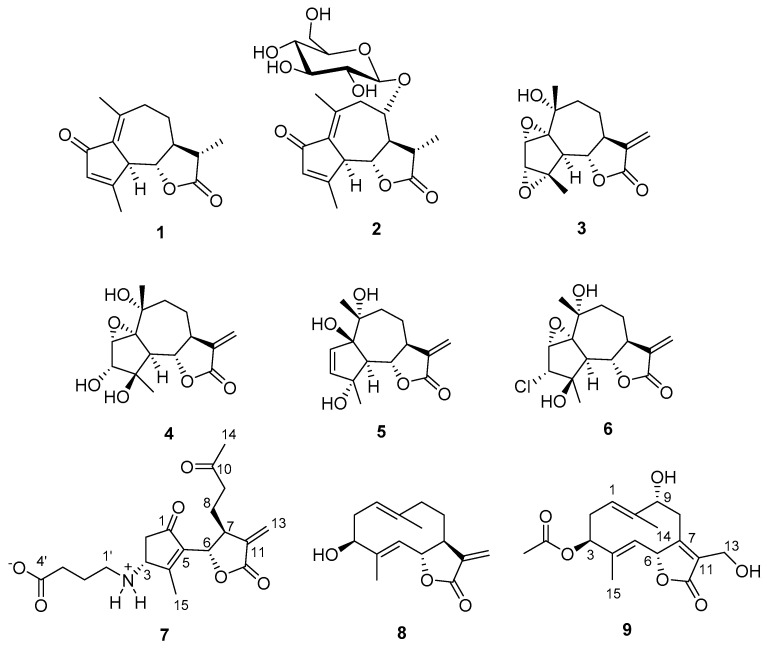
Sesquiterpenoid constituents of *A. wilhelmsii*.

**Figure 2:**
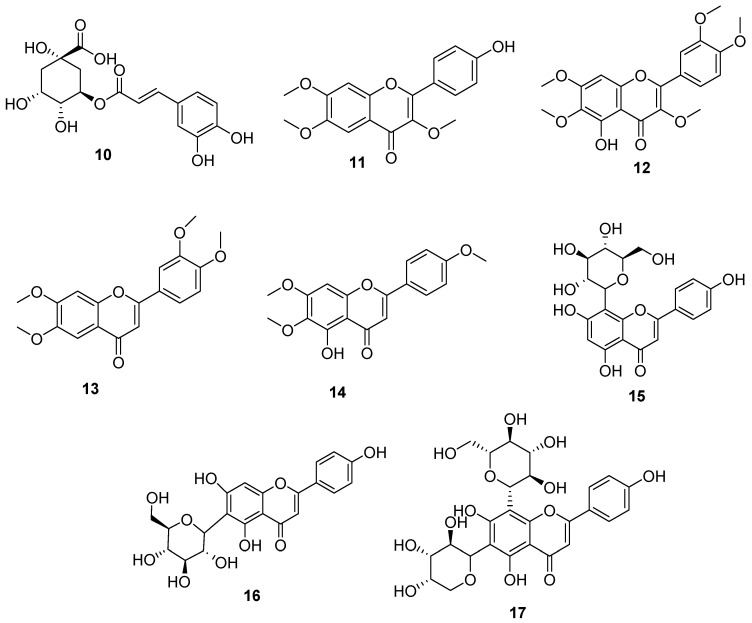
Phenolic constituents of *A. wilhelmsii*.

A comprehensive phytochemical investigation of these three extracts, involving chromatography on silica and RP18 phases and HPLC purifications, resulted in the isolation and subsequent structural characterization of 17 secondary metabolites in the pure state. Nine of these compounds (Figure 1, **1**–**9**) were characterized as sesquiterpenoids belonging to the guaiane, seco-guaiane, and germacrane skeletons and included two new metabolites, named wilhelmsin (**7**) and wilhelmsolide (**9**). The known leukodin (**1**) [12], deacetylmatricarin-8-*O*-*β*-glucopyranoside (**2**) [13], artecanin (**3**) [14], artecaninhydrate (**4**) [15], artemargyinolide B (**5**) [16], chloroklotzchin (**6**) [17], and hanphyllin (**8**) [18] were identified on the basis of the comparison of their spectral data with those reported in the literature [12,13,14,15,16,17,18]. Compounds **2** and **5** had never been reported before from plants of the genus *Achillea.* The remaining eight compounds (Figure 2) were phenolic derivatives, identified as chlorogenic acid (**10**) [19], the polymethoxylated flavonols penduletin (**11**) [20] and artemetin (**12**) [21], the polymethoxylated flavones santoflavone (**13**) [22] and salvigenin (**14**) [23] and the flavone-*C*-glycosides vitexin (**15**) [24], isovitexin (**16**) [25], and isoschaftoside (**17**) [26].

**Figure 3:**
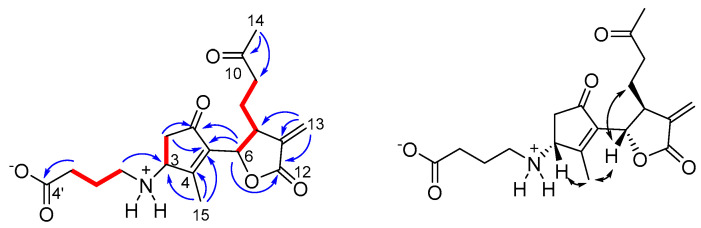
(**Left**): COSY (red bold) and key HMBC (blue arrows) correlations of **7.** (**Right**): ROESY correlations of **7**.

Wilhelmsin (**7**) was isolated as an optically active colorless amorphous solid with molecular formula C_19_H_25_NO_6_, as determined by HR-ESIMS. The ^1^H NMR spectrum of **7** (CD_3_OD) (Table 1 and Figures S1–S7) showed signals for a *sp^2^* methylene (δ_H_ 6.26 and 5.75), two resonances in the midfield region (δ_H_ 5.08 and 5.22), a series of multiplets located between δ_H_ 3.38 and 1.87 and two relatively deshielded methyl singlets at δ_H_ 2.19 and 2.05. All the proton signals were associated to those of the corresponding ^13^C NMR signals through the 2D NMR HSQC spectrum. The 2D NMR COSY experiment allowed us to identify three spin systems (Figure 3) including all the multiplets of the ^1^H NMR spectrum. ^2,3^*J*_C,H_ correlations of the 2D NMR HMBC experiment were instrumental to join the above fragments and disclose the planar structure of wilhelmsin (**7**). In particular, correlations from the *sp^2^* methylene signals H_2_-13 to C-7, C-11 and the ester C-12 (δ_C_ 170.8), combined to the correlation H-6/C-12 defined the presence of a substituted exomethylene-γ-lactone ring. Similarly, correlations of the allylic methyl H_3_-15 (δ_H_ 2.05) to C-3, C-4, and C-5 and of H_2_-2 to the ketone carbonyl C-1 (δ_C_ 203.7) and to C-5 identified the presence of a cyclopentenone, for which correlations H-6/C-5 and H-6/C-1 indicated direct linkage to the lactone ring. Correlations of H_3_-14 to the lactone C-10 (δ_C_ 208.9) and to C-9 defined the structure of the 4C side chain attached at C-7. The substituent attached at C-3 was defined by the HMBC correlations H_2_-1′/C-3 and H_2_-3′/C-4′. The ^13^C NMR resonance of C-1′ (δ_C_ 42.8) fully supported the placement of the nitrogen atom indicated by the molecular formula. The relative configuration at the three stereogenic centers of **7** was defined from the ROESY correlations of H-6 with H_2_-8 and of H_3_-15 with both H-3 and H-6 (Figure 3).

Wilhelmsin (**7**) is a new member of the class of 1,10-*seco*-guaianolide derivatives, which was not yet described in *Achillea* plants and in other Asteraceae. Almost invariably, these compounds bear an -OH or an -OMe group at C-3, as a result of the strongly electrophilic nature of this carbon. To our knowledge, wilhelmsin is the first compound of this class, and more generally of sesquiterpene-amino acid adducts, to include the non-protein amino acid γ-aminobutyric (GABA) in its structure. Interestingly, it has been found that GABA rapidly accumulates in plant tissues in response to biotic and abiotic stress and regulates plant growth via aluminum-activated malate transporter (ALMT) proteins [27].

**Figure 4:**
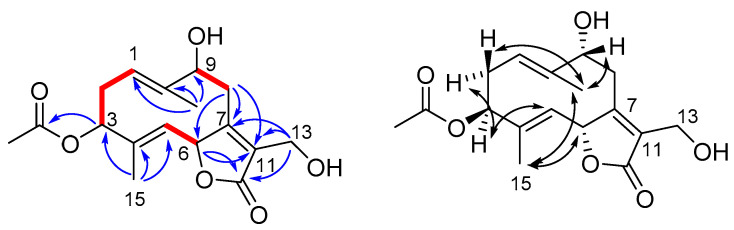
(**Left**): COSY (red bold) and key HMBC (blue arrows) correlations of **9.** (**Right**): ROESY correlations of **9**.

Wilhelmsolide (**9**) was determined to be C_17_H_22_O_6_ by HR-ESIMS, implying seven degrees of unsaturation. The ^1^H NMR spectrum of **9** (CDCl_3_) (Table 2 and Figures S1–S7) showed signals of three methyl singlets (H_3_-14 and H_3_-15), including one acetyl methyl (δ_H_ 2.10). The other signals were arranged into three spin systems (Figure 4) upon interpretation of COSY correlations and, after association proton-carbon signals through the HSQC spectrum, the HMBC signals could allow the assembly of the above fragments. In particular, correlations from the two allylic methyl singlets H_3_-14 and H_3_-15 and those of H_2_-8 (Figure 4) defined the architecture of the dimethylated and trioxygenated ten-membered ring. On the other hand, correlations from H-6 to C-11 and the ester C-12 and from the diastereotopic methylene H_2_-13 to C-12 and to the *sp^2^* C-7 and C-11 defined the structure of the α,β-unsaturated γ-lactone ring bearing an hydroxymethyl group at position α. The acetyl moiety was confidently attached at the 3-OH on the basis of the low-field value of H-3 (δ_H_ 5.14) and the HMBC cross-peak of H-3 with the ester carbonyl. The geometry of the double bonds Δ^1,2^ and Δ^4,5^ and the relative configuration of the three stereogenic centers H-3, H-6, and H-9 in the germacranolide structure of wilhelmsolide (**9**) could be deduced by the network of ROESY correlations summarized in Figure 4.

**Figure 5:**
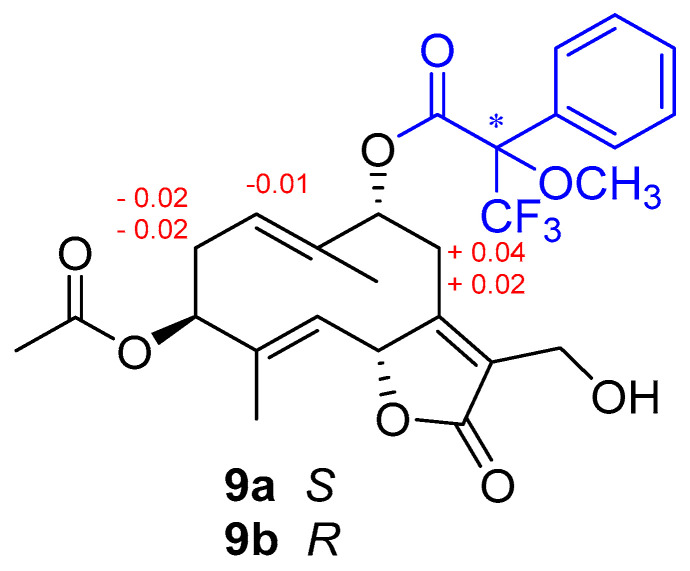
Application of the Mosher’s method to wilhelmsolide (**9**). Selected Δδ_(S-R)_ values (in ppm) are shown.

Taking advantage of the presence of an oxymethine in the structure of wilhelmsolide (**9**), the relative configuration was upgraded to the absolute one by application of the modified Mosher’s method for secondary alcohols [28]. Thus, compound **9** was treated with R-(–)- and S-(+)-MTPA chloride to give the S-MTPA (**9a**) and R-MTPA (**9b**) ester, respectively (Figure 5). Analysis of the Δδ_(S-R)_ values, following the Mosher’s model [29] suggested the assignment of *R* configuration at C-9 of wilhelmsolide (Figure 5). Given the above-determined relative arrangement, the absolute configuration of the entire molecule was inferred.

### 3.2. Biological Evaluation

To identify *A. wilhelmsii* secondary metabolites responsible for the effects on metabolic syndrome ascribed to the plant extract, we used different screening pipelines, aiming at the selection of molecules active either in reducing cholesterol and fatty acid biosynthesis and/or in improving glucose homeostasis and mitochondrial activity.

#### 3.2.1. Lipid Lowering Activity

**Figure 6:**
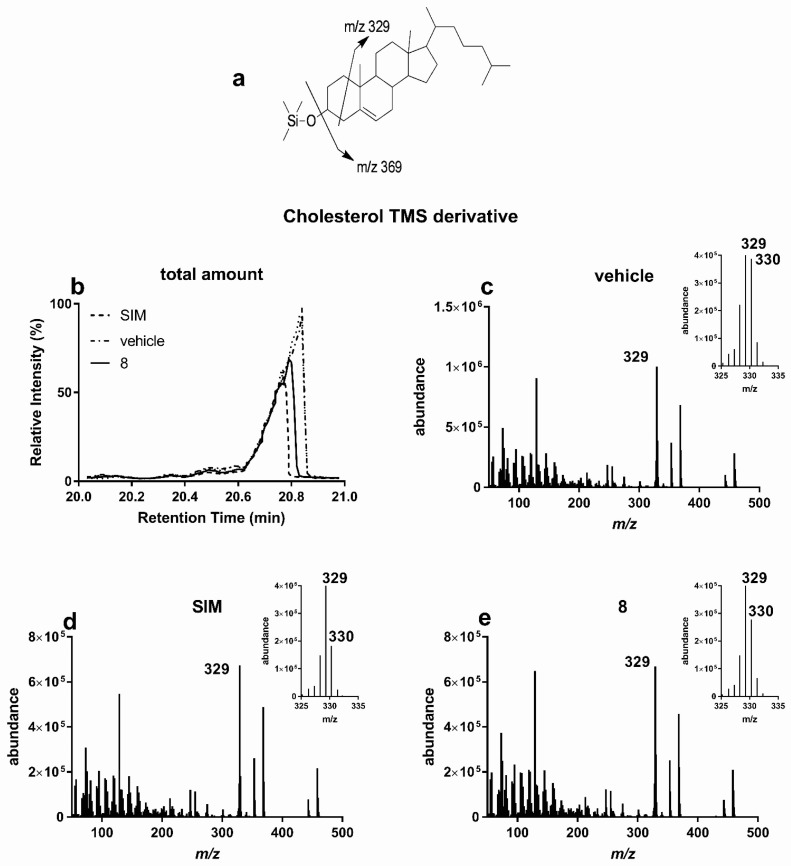
Hanphyllin (**8**) inhibits cholesterogenesis in HuH7 cells. HuH7 cells were grown in the presence of D_2_O and treated for 72 h in the presence of SIM, **8** or the corresponding volume of vehicle (DMSO). (**a**) fragmentation pattern of cholesterol TMS derivative; (**b**) comparison of gas chromatography-mass spectrometry (GC/MS) spectra (intensity versus retention time) of samples extracted from HuH7 cells treated with hanphyllin, vehicle, or SIM. (**c**–**e**) MS analysis of GC peaks eluting at 20.8 min and containing undeuterated TMS derivatized cholesterol (*m/z* 458 M–H_2_0), a cholesterol fragment (*m/z* 329 M-H_2_0–C_8_H_16_), and their corresponding deuterated forms (see corresponding enlarged insets). (Representative of at least three experiments).

Cholesterol and fatty acid biosynthesis are anabolic reactions necessary for cellular homeostasis. However, these are hyperactivated in dysmetabolic conditions and usually lead to cholesterol deposition in vascular endothelia, atherosclerosis, triglyceride accumulation, steatosis, and liver diseases. In vitro growing hepatic HuH7 cells were labelled with D_2_O and cultured in the presence of the tested compounds for 72 h (Table 3). In virtue of its activity against cholesterogenesis, simvastatin (SIM, 30 μM) was used as positive control molecule. Upon extraction in the organic solvent, cellular lipids were derivatized with TMS and analyzed by GC/MS (Figure 6). Among the tested metabolites and similarly to SIM, cells treated with hanphyllin (**8**) showed lower amount of total cholesterol compared to untreated HuH7 cells (Figure 6a,b). To highlight any effect of hanphyllin on cholesterogenesis, we measured the amount of deuterium incorporated in newly synthesized cholesterol molecules. Chemical species endowed with molecular masses heavier than those naturally occurring (mainly Δ*m/z* =1–2 Da) were found co-eluting with molecular ions and their fragments. The presence of these heavier species in untreated cells indicates that cholesterogenesis was occurring in HuH7 cells (Figure 6c). As expected, when HuH7 cell were cultured in the presence SIM (Figure 6d), *m/z* peaks corresponding to deuterated cholesterol molecules were all decreased. Treatment with hanphyllin resulted in a reduction of cholesterol deuterated peaks, proving that, such as SIM, its cholesterol lowering activity involves inhibition of de novo cholesterogenesis (Figure 6e). A similar reduction in cholesterol biogenesis was obtained upon incubation with santoflavone (**13**).

**Figure 7:**
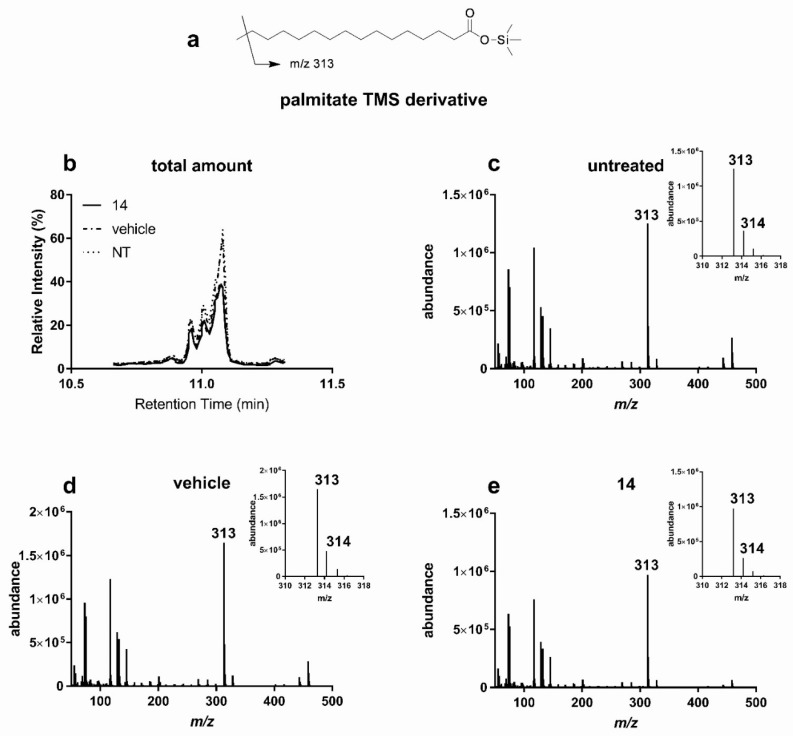
Salvigenin (**14**) inhibits palmitic acid biosynthesis in HuH7 cells. HuH7 cells were grown in the presence of D_2_O and treated for 72 h in the presence of salvigenin or the corresponding volume of vehicle (DMSO). (**a**) fragmentation pattern of palmitic acid TMS derivative; (**b**) comparison of gas chromatography-mass spectrometry (GC/MS) spectra (intensity versus retention time) of samples extracted from HuH7 cells treated with salvigenin (**14**), vehicle, or left untreated. (**c**–**e**) MS analysis of GC peaks eluting at 11.1 min and containing undeuterated TMS derivatized palmitic fragment (*m/z* 313 M-CH_3_), and its corresponding deuterated forms (*m/z* 314 and 315 see corresponding enlarged insets). (Representative of at least three experiments).

Next, we used GC/MS to measure the intracellular levels of palmitic acid, one of the most abundant lipids of biological membranes, (Figure 7). In virtue of its lipogenesis inhibitory activity, apple polyphenolic extracts (APE, 400 mg/L) [10] was used as positive control. Compared to cells left untreated or treated with vehicle, incubation with hanphyllin (**8**) did not exert any effect on the total amount of fatty acid present in the cell (Table 3). However, we did measure a reduction of palmitic acid (Figure 7a,b) in cells treated with santoflavone (**13**) and salvigenin (**14**), suggesting their effect on fatty acid homeostasis. The rate of deuterium incorporation in newly synthesized palmitic acid molecules confirmed that the palmitate lowering activity of the two flavone derivatives involves inhibition of de novo lipogenesis. Indeed, compared to cells left untreated (Figure 7c) or treated with vehicle (Figure 7d), HuH7 treated with compound salvigenin (**14**) presented a reduction in the intensity of palmitic acid deuterated *m/z* peaks (Figure 7e).

**Figure 8:**
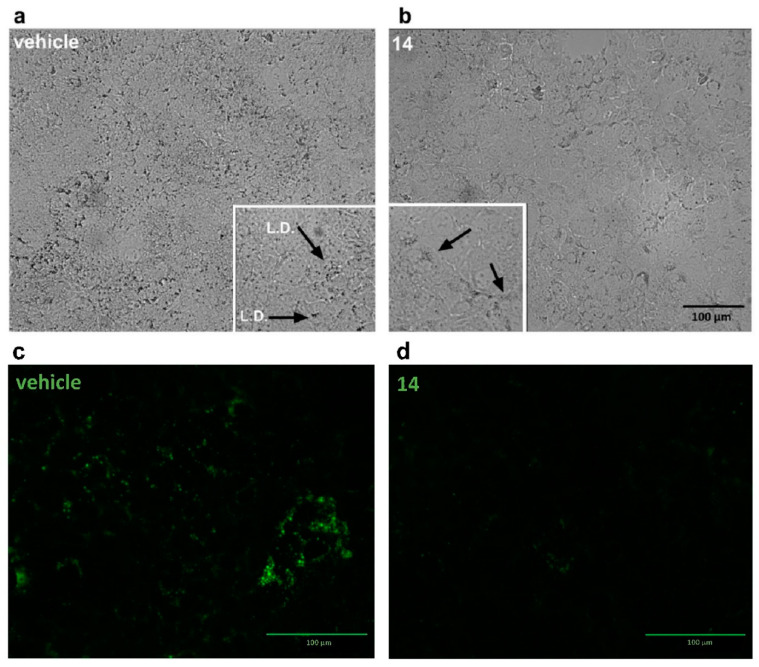
Salvigenin (**14**) reduces lipid droplets in HuH7 cells. HuH7 cells were grown in the presence of salvigenin (**14**) (30 μM) or the corresponding volume of vehicle (DMSO). Lipid droplets, visible as electron-dense material in the cytoplasm of HuH7 cells (**a**,**b**) or upon staining with the lipid tracer BODIPY 493/503 (**c**,**d**), are reduced upon treatment with (see enlarged insets and cytoplasmic structures pointed by the arrows and stained by the dye). (Representative of at least three experiments). Magnification bar = 100 μm.

The inhibitory effect on lipogenesis of salvigenin (**14**) was also visible using immunofluorescence as well as light microscopy. HuH7 cells present intracytoplasmic lipidic droplets (containing mostly triglycerides) that appear electron-dense (Figure 8a,b) and are stained by BODIPY 493/503, a fluorescent dye staining neutral lipids such as triglycerides (Figure 8c,d). HuH7cells treated with compound **14** showed a drastic reduction in the number of droplets present intracellularly, confirming the inhibitory activity of the compound toward lipogenesis (Figure 8).

#### 3.2.2. Mitochondrial Stimulatory Activity

In a second pipeline, we evaluated the effect of *A. willhelmsii* metabolites on the mitochondrial activity of in vitro growing HuH7 cells. Mitochondrial activity was assessed using the mitochondrial selective probe MitoTracker CMXRos. This probe accumulates in the intermembrane space of mitochondria and emits fluorescence with an intensity that positively correlates with the difference in potential existing between the mitochondrial matrix and the mitochondrial intermembrane space.

This electrochemical potential reflects the activity of the H^+^ pumps of the electron transport chain involved in oxidative respiration. In virtue of its mitochondrial stimulatory activity, apple polyphenolic extracts (APE, 400 mg/L) [10] was used as positive control. Among the tested metabolites (Table 3), cells treated with salvigenin (**14**) presented the highest mitochondrial activity.

#### 3.2.3. Glucose Uptake Modulatory Activity

The third pipeline assessed the ability of *A. willhelmsii* metabolites to modulate glucose uptake via glucose transporter (GLUT). Handling of circulating glucose levels is compromised in dysmetabolic syndromes, that are often characterized by insulin resistance and hyperglycemia. GLUTs are a family of transporters playing a pivotal role in the uptake of glucose from circulation. Most of them are regulated by insulin receptors via PI3K signaling and respond to insulin stimulation. Insulin stimulation can either increase the number of GLUT receptors (mainly GLUT4) localized on the plasma membrane of the cells as well as promote glycolysis and ultimatelly increase the activity of passive transporters (GLUT1) by decreasing the amount of intracellular glucose. GLUT transporter activity is compromised in condition of Insulin resistance and their pharmacological activation improves glucose tolerance and ameliorates hyperglycemia. Glucose uptake was assessed by measuring the uptake of NBDG, a fluorescent analogue of deoxy-glucose covalently linked to the fluorescent molecule nitro blue tetrazolium (NBT). The uptake of NBDG increases the fluorescence of living cells indicating the activity of the GLUT transporters. HuH7 cells promptly responds to an insulin short stimulation by augmenting glucose uptake. Among the tested metabolites (Table 3), cells treated with santoflavone (**13**) were the only presenting increased glucose uptake compared to control cells.

## 4. Discussion

The metabolic syndrome refers to the co-occurrence of several known cardiovascular risk factors, including insulin resistance, obesity, atherogenic dyslipidemia, and hypertension. The syndrome is posing substantial concern since it has reached epidemic proportions worldwide. It is widely recognized that botanicals may serve as effective agents for the prevention or treatment of metabolic syndrome since they contain biologically active secondary metabolites that, by exerting multiple mechanisms of action, may potentiate each other’s activity, or have a synergistic effect [29]. Although the synergistic effect of structurally related (or sometimes even unrelated) metabolites plays a crucial role in the activity of plant extracts, a detailed knowledge of the chemical composition and of the biological activity of each single compound is nevertheless required. Indeed, it could allow the optimization of plant extraction, the standardization of extracts or the preparation of extracts enriched in one class of compounds.

We have applied these principles to *A. wilhelmsii*, a plant consumed as a hot beverage in the Middle East region and for its claimed beneficial effects in different conditions, such as treatment of oxidative stress-related myocardial injury [7] or lowering plasma triglycerides and LDL-cholesterol [8,9]. It is known that *A. wilhelmsii* elaborates mainly sesquiterpenoids and phenolic compounds, with special regards to methoxylated flavones and flavone-*C*-glycosides, but a comprehensive characterization of both these classes of compounds was still lacking. In the present investigation, we have obtained in the pure state and chemically characterized 17 compounds from different extractions of the plant (nine sesquiterpenoids and eight phenolic derivatives), including the new sesquiterpene derivatives wilhelmsin (**7**) and wilhelmsolide (**9**). Wilhelmsin is characterized by a *seco*-guaianolide skeleton conjugated to a GABA moiety, a feature not previously known.

We isolated 17 pure compounds and tested them for their cholesterol/fatty acid lowering activity and effects on glucose homeostasis, both hallmarks of beneficial effects against metabolic syndrome. The sesquiterpene hanphyllin (**8**) showed a marked effect in the reduction of cholesterol biosynthesis, with no effect in other bioactivities tested. In our in vitro assay, at the dose of 30 μM compound **8** reduced cholesterogenesis with a potency similar to the statin Simvastatin (12.2% versus 14.8% decrease in cholesterol biosynthesis for **8** and Simvastatin, respectively). Some naturally occurring sesquiterpenoids of the guaianolide and germacranolide classes have already been reported to exert antihyperlipidemic activities, but in most cases their activity is not selective [30]. It has been argued that their activity could be ascribed to electrophilic reactivity toward thiol-bearing enzymes of lipid synthesis, i.e., citrate-lyase, acetyl-CoA synthetase, and beta-hydroxy-beta-methylglutaryl-CoA reductase, with a Michael-type mechanism [30]. In this regard, the α-methylene-γ-lactone moiety should be hypothesized as the pharmacophoric moiety responsible for this activity. The activity of hanphyllin does not disprove this hypothesis, but it must be noted that a similarly (if not more) reactive moiety is also present in compounds **3–7,** thus indicating that the overall skeleton of the compound plays a crucial role in the adaptation to their target enzymes’ active site. Indeed, it can be noticed that hanphyllin is the single germacranolide derivative, while **3–7** are all guaianolides. 

As mentioned before, hanphyllin did not exert effects on fatty acid homeostasis, while the tetra- and tri-methoxylated flavones santoflavone (**13**) and salvigenin (**14**) showed marked palmitate lowering activity, with a drastic reduction in the number of lipid droplets present intracellularly, via inhibition of de novo lipogenesis. Moreover, in this case, there are reports in the literature about the beneficial effect of flavones in reducing lipid levels in hyperlipidemic rats [31], however, our study updates the available information by showing that: (a) free -OH groups are not essential for activity since polymethoxylated flavones are also very active; (b) the flavone structure is not sufficient for activity since strict structural requirements appear to be operating. Compared to santoflavone (**13**), the most active flavone salvigenin (**14**) possesses an -OH group on ring A which is clearly beneficial for activity. On the contrary, the presence of a methoxy group on ring B of the flavonol artemetin (**12**) plays a dramatically negative effect on bioactivity. Interestingly, santoflavone (**13**) was active in stimulating glucose uptake via GLUT transporter. Further investigation will be necessary to confirm the mechanism of action of this molecule and if it either affects GLUT transporters, insulin receptors, or indirectly promote intracellular catabolism and glucose uptake via GLUT independent transporters. 

Mitochondria are master regulators of cellular functions, and their reduced activity is linked to dysmetabolic conditions, while pharmacological stimulation of mitochondria by polyphenols [10] and other plant metabolite can accelerate intracellular catabolic reactions with beneficial effects on metabolic syndrome. Indeed, an accelerated catabolism may promote lipolysis, glucose uptake, and inhibition of lipid and cholesterol biosynthesis in hepatic cells as well as in adipose tissue.

Salvigenin (**14**) was the single *A. wilhelmsii* metabolite to show a significant mitochondrial stimulatory activity, measured as an increase in the activity of the H^+^ pumps of the electron transport chain involved in oxidative respiration. The exact mechanism of this activity is not known and could be related either to a direct effect on enzymes of mitochondrial complexes or to a modulation of transcription factors which regulate the expression of mitochondrial proteins. The result of this activity is a complex balance between a pro-oxidant metabolism-stimulating activity (with the consequent increase in ROS production) and the antioxidant effect at molecular level associated to flavones and related flavonoids. Overall, the dual activity detected for salvigenin (**14**) on both lipogenesis and mitochondrial functionality could suggest a link between the two processes. Most likely, the mitochondrial stimulatory activity stimulates fatty acid β-oxidation, with this latter serving as fuel for oxidative phosphorylation and ATP production in mitochondria.

## 5. Conclusions

A comprehensive phytochemical analysis on aerial parts of the Iranian plant *A. wilhelmsii* has led to the isolation of 17 pure secondary metabolites belonging to the classes of sesquiterpenoids and phenolics. Two of these compounds, named wilhemsin (**7**) and wilhelmsolide (**9**), are new sesquiterpenoids, the first showing unprecedented structural features. All the pure compounds were evaluated on endpoints related to the treatment of metabolic syndrome to investigate an effect previously reported for the plant extracts. The sesquiterpene hanphyllin (**8**) showed a selective cholesterol-lowering activity, the santoflavone (**13**) improves glucose transport, while the trimethoxylated flavone salvigenin (**14**) showed a dual activity in decreasing lipid levels and stimulating mitochondrial functionality.

Overall, our study further confirms that, in addition to demonstrated antioxidants such as vitexin [32], isovitexin, and isoschaftoside, *A. wilhelmsii* extracts contain molecules that can act at different levels on the metabolic syndrome symptoms, suggesting that their combined and synergistic action could be a valid strategy, worthy of being further investigated and exploited.

## Figures and Tables

**Table 1:** ^1^H (600 MHz) and ^13^C (150 MHz) NMR Data of wilhelmsin (**7**) in CD_3_OD.

Pos.	δ_H,_ Multiplicity, *J* in Hz	δ_C,_ Type
1		203.7, C
2a	2.69–2.71	37.2, CH_2_
2b	2.44–2.45	
3	5.22, br d, 6.5	53.5, CH
4		171.4, C
5		139.1, C
6	5.08, d, 5.4	76.3, CH
7	3.19, overlapped	42.7, CH
8a	1.94–1.96	27.3, CH_2_
8b	1.86–1.88	
9	2.59, t, 7.3	39.2, CH_2_
10		208.9, C
11		139.0, C
12		170.8, C
13a	6.26, d, 2.8	121.3, CH_2_
13b	5.75, d, 2.8	
14	2.19, s	28.5, CH_3_
15	2.05, s	12.8, CH_3_
	3.38–3.39	42.8, CH_2_
1′	3.10, overlapped	
2′	2.09, overlapped	17.5, CH_2_
3′	2.45, overlapped	30.7, CH_2_
4′		177.1, C

**Table 2:** ^1^H (400 MHz) and ^13^C (100 MHz) NMR Data of wilhelmsolide (**9**) in CDCl_3_.

Pos.	δ_H,_ Multiplicity, *J* in Hz	δ_C,_ Type
1	4.99, dd, 2.5, 12.1	125.8, CH
2a	2.54–2.56	30.1, CH_2_
2b	2.35–2.36	
3	5.14, dd, 6.1, 10.3	77.8, CH
4		137.4, C
5	4.38, d, 10.5	122.6, CH
6	5.44, d, 10.5	80.6, CH
7		161.9, CH
8a	2.98, dd, 13.8, 1.5	35.0, CH_2_
8b	2.60, dd, 10.1, 13.8	
9a	4.27, dd, 10.1, 1.5	78.1, CH
10		139.5, C
11		129.7, C
12		172.9, C
13a	4.48, d, 14.1	55.2, CH_2_
13b	4.54, d, 14.1	
14	1.64, s	10.5, CH_3_
15	1.74, s	12.0, CH_3_
AcO-	2.10, s	21.1, CH_3_ 170.1, C

**Table 3:** Effect of *A. wilhelmsii* compounds (30 μM) on metabolic activity.

Compound	Cholesterol Biosynthesis ^a^	Palmitic Acid Biosynthesis ^b^	Mitochondrial Activity ^c^	Glucose Uptake ^d^
vehicle	–8.3 ± 0.6 **	2.5 ± 0.2 *	–0.3 ± 0.6 ^n.s^	0.4 ± 0.2 ^n.s^
Simvastatin	14.8 ± 4.0 ***	-	-	-
A.P.E. (400 mg/L)	-	–47.6 ± 1.9 ***	-	-
A.P.E. (400 mg/L)	-	-	22.6 ± 0.9 ***	-
Insulin (100 nM)	-	-	-	74.4 ± 3.6 ***
**1**	−7.3 ± 0.2 *	0.7 ± 1.1 ^n.s.^	2.1 ± 0.2 ***	0.9 ± 1.2 ^n.s.^
**2**	−8.1 ± 0.4 **	–1.9 ± 0.3 ^n.s.^	0.3 ± 0.3 ^n.s.^	1.9 ± 0.4 ^n.s.^
**3**	−8.1 ± 2.2 **	0.1 ± 1.7 ^n.s^	2.4 ± 0.2 ***	0.3 ± 0.5 ^n.s.^
**4**	−9.1 ± 3.0 *	1.2 ± 0.2 ^n.s.^	3.7 ± 0.6***	0.9 ± 0.6 ^n.s.^
**5**	−10.1 ± 2.2 *	–1.6 ± 2.1 ^n.s.^	0.5 ± 0.2 ^n.s.^	0.4 ± 0.7 ^n.s.^
**6**	–17.4 ± 7.4 *	–1.5 ± 0.2 ^n.s.^	1.9 ± 0.5 ***	1.7 ± 1.2 ^n.s.^
**7**	–19.9 ± 4.1 *	2.1 ± 1.2 ^n.s.^	2.6 ± 0.3 ***	0.8 ± 0.7 ^n.s.^
**8**	–12.7 ± 0.1 ***	2.9 ± 1.7 *	1.8 ± 0.2 ***	1.4 ± 0.4 ^n.s.^
**12**	-8.6 ± 3.2 *	0.9 ± 1.2 ^n.s.^	0.2 ± 0.5 ^n.s.^	0.7 ± 0.8 ^n.s^
**13**	–12.2 ± 2.2 **	–6.3 ± 0.4 ***	1.6 ± 0.2 ***	16.2 ± 0.2 ***
**14**	–9.1 ± 1.6 *	–22.5 ± 0.7 ***	15.4 ± 0.4 ***	0.7 ± 0.5 ^n.s^
**15**	–8.1 ± 0.9 *	1.0 ± 0.5 ^n.s^	0.8 ± 0.6 ^n.s.^	1.4 ± 0.9 ^n.s^
**16**	–5.7 ± 2.2 ^n.s.^	2.3 ± 1.5 ^n.s.^	2.2 ± 0.3 ***	0.6 ± 1.9 ^n.s^
**17**	–6.9 ± 2.9 *	1.9 ± 1.3 ^n.s.^	1.9 ± 0.5 ***	1.5 ± 0.5 ^n.s^

HuH7 cells were grown in the presence of the indicated compounds or the corresponding volume of vehicle (DMSO). Simvastatin, apple polyphenolic extract [10], Insulin were used as positive controls for each assay. ^(a)^ Rate of deuterium incorporation in newly synthesized cholesterol molecules expressed as percentage of difference in cholesterol biosynthesis compared to untreated samples; ^(b)^ rate of deuterium incorporation in newly synthesized palmitic acid molecule expressed as percentage of difference in palmitic acid biosynthesis compared to untreated samples (*n* = 3; results are expressed as mean ± s.d.); ^(c)^ variation in mitochondrial difference in potential expressed as percentage of increase in mitochondrial activity compared to untreated samples (*n* = 3, results are expressed as mean ± s.d.); ^(d)^ increase in glucose uptake via GLUT transporters expressed as percentage of glucose uptake compared to untreated samples (*n* = 3, results are expressed as mean ± s.d.)**;** *p* value * < 0.01, ** < 0.05, *** < 0.001; n.s. indicates that the effect of the compound is not statistically different from that measured in untreated cells.

## Data Availability

Data is contained within the article and Supplementary Material.

## Supplementary Materials

The following are available online at https://www.mdpi.com/article/10.3390/antiox10071042/s1, Figures S1–S7: 1D and 2D NMR spectra of wilhelmsin and wilhelmsolide.
