# Supplementary material for: Salvigenin, a Trimethoxylated Flavone from Achillea Wilhelmsii C. Koch, Exerts Combined Lipid-Lowering and Mitochondrial Stimulatory Effects
Source: Antioxidants (Basel). 2021 Jun 29;10(7):1042. doi: 10.3390/antiox10071042 (PMC8300625; doi:10.3390/antiox10071042)
Supplement: Supplementary file 1 [file antioxidants-10-01042-s001.zip › antioxidants-1209288-supplementary.pdf]

SUPPLEMENTARY MATERIALS

Salvigenin, a trimethoxylated flavone from *Achillea wilhelmsii* C. Koch, Exerts Combined Lipid-Lowering and Mitochondrial Boosting Effects

Elena Serino[1,#], Azam Chahardoli[2,#], Nadia Badolati[1], Carmina Sirignano[1], Fereshteh Jalilian[2], Mahdi Mojarrab[2], Zahra Farhangi[3], Daniela Rigano[1], Mariano Stornaiuolo[1,*], Yalda Shokoohinia[2,4,*], and Orazio Taglialatela-Scafati[1,*]

[1]Department of Pharmacy, School of Medicine and Surgery, University of Naples Federico II, Via Montesano 49, 80131 Naples, Italy; e.serino96@mail.com (E.S.); nadia.badolati@unina.it (N. B.); carmina.sirignano@unina.it (C.S.) ; drigano@unina.it (D. R.).

[2]Pharmaceutical Sciences Research Center, Kermanshah University of Medical Sciences, 6715847141 Kermanshah, Iran; a.chahardoly@gmail.com (A. C.); fe.jalilian@gmail.com (F. J.); mahdi.mojarab@gmail.com (M. M.).

[3]Student Research Committee, Kermanshah University of Medical Sciences, 6715847141 Kermanshah, Iran; farhangi_zr@yahoo.com (Z. F.).

[4]Ric Scalzo Institute for Botanical Research, Southwest College of Naturopathic Medicine, Tempe, AZ 85282, USA.

*Correspondence: mariano.stornaiuolo@unina.it, +39-081678117 (M.S.); y.shokoohinia@scnm.edu , +1-480- 858-9100 (Y.S.); scatagli@unina.it, +39-081678509 (O.T.S.).

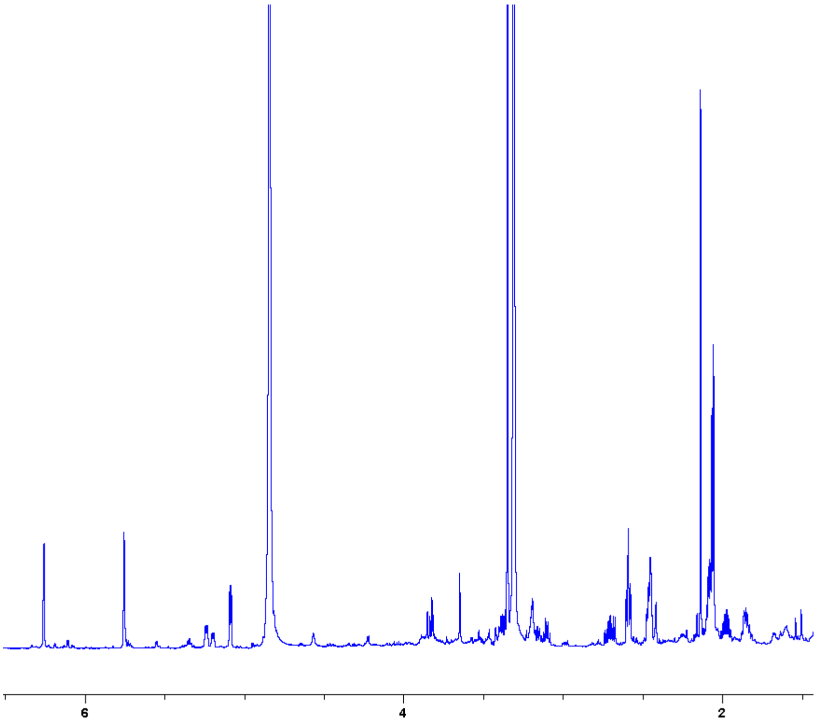

Figure S1. ^{1}H NMR spectrum of wilhelmsin (CD_3OD)

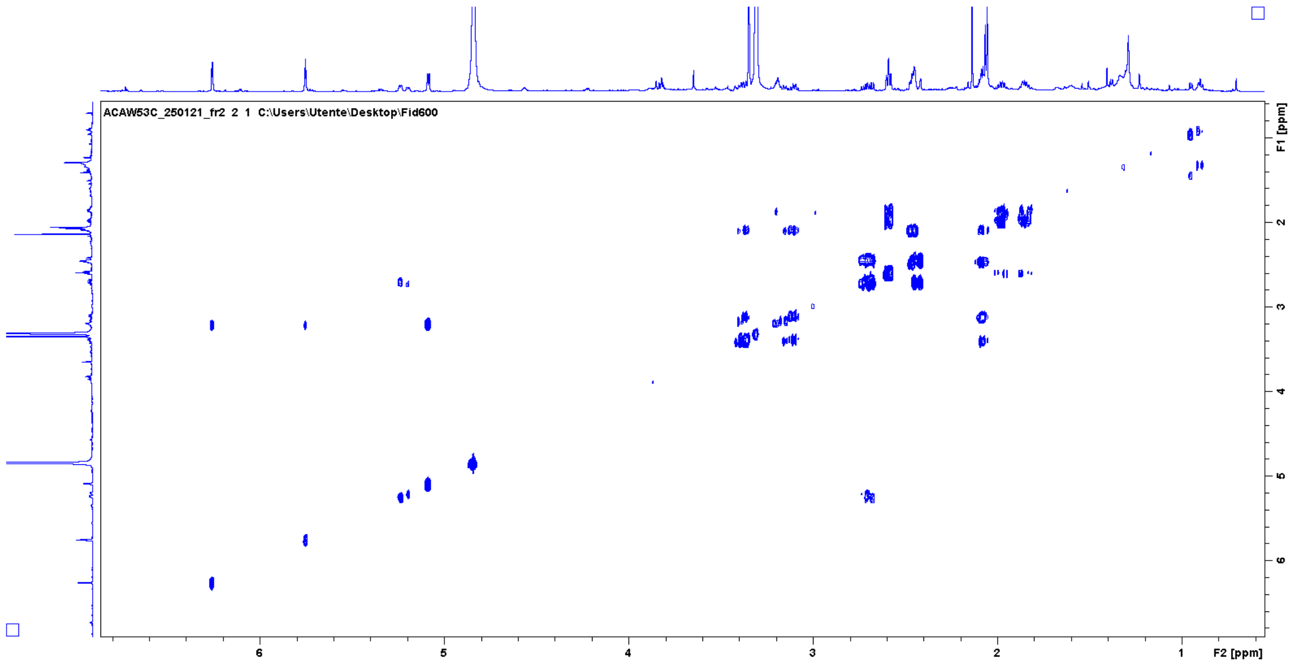

Figure S2. 2D COSY NMR spectrum of wilhelmsin (CD_3OD)

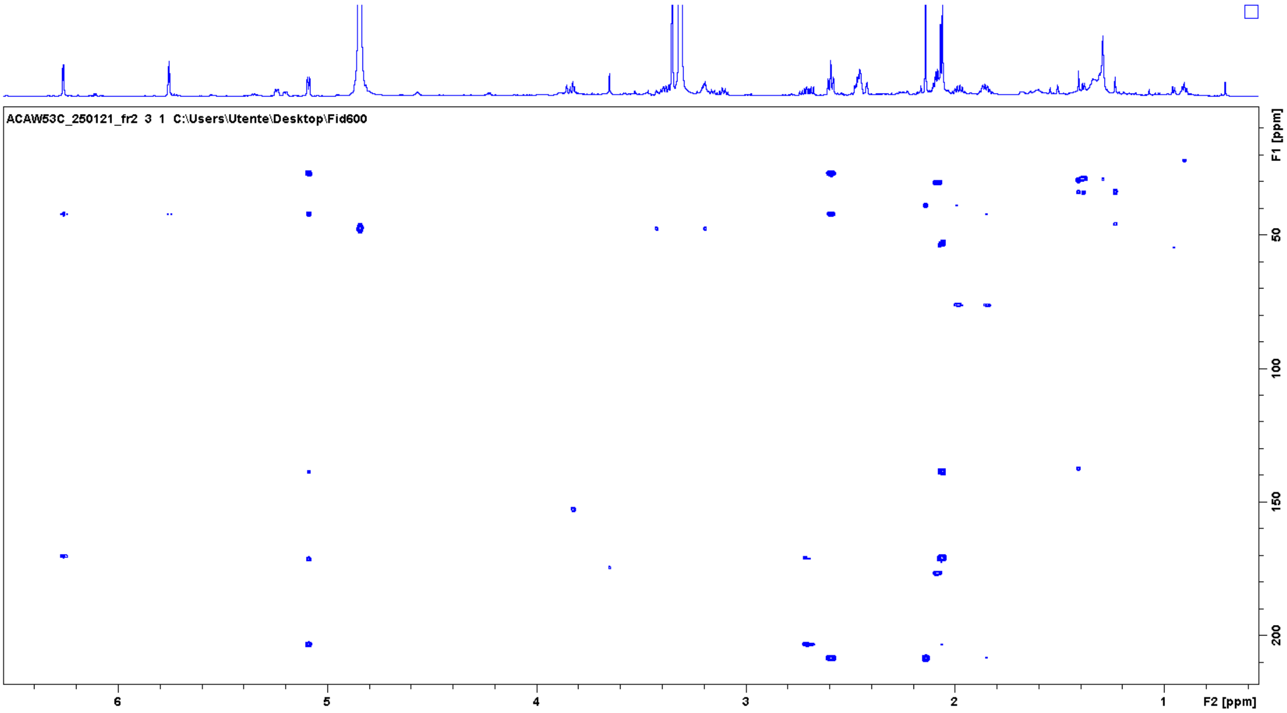

Figure S3. 2D HMBC NMR spectrum of wilhelmsin (CD_3OD)

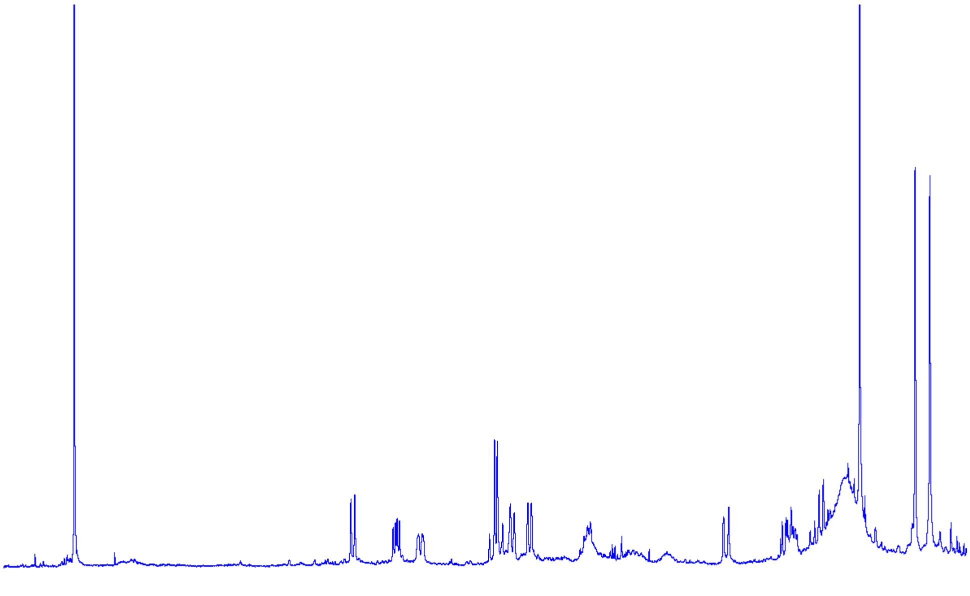

Figure S4. ^1H NMR spectrum of wilhelmsolide ($CDCl_3$)

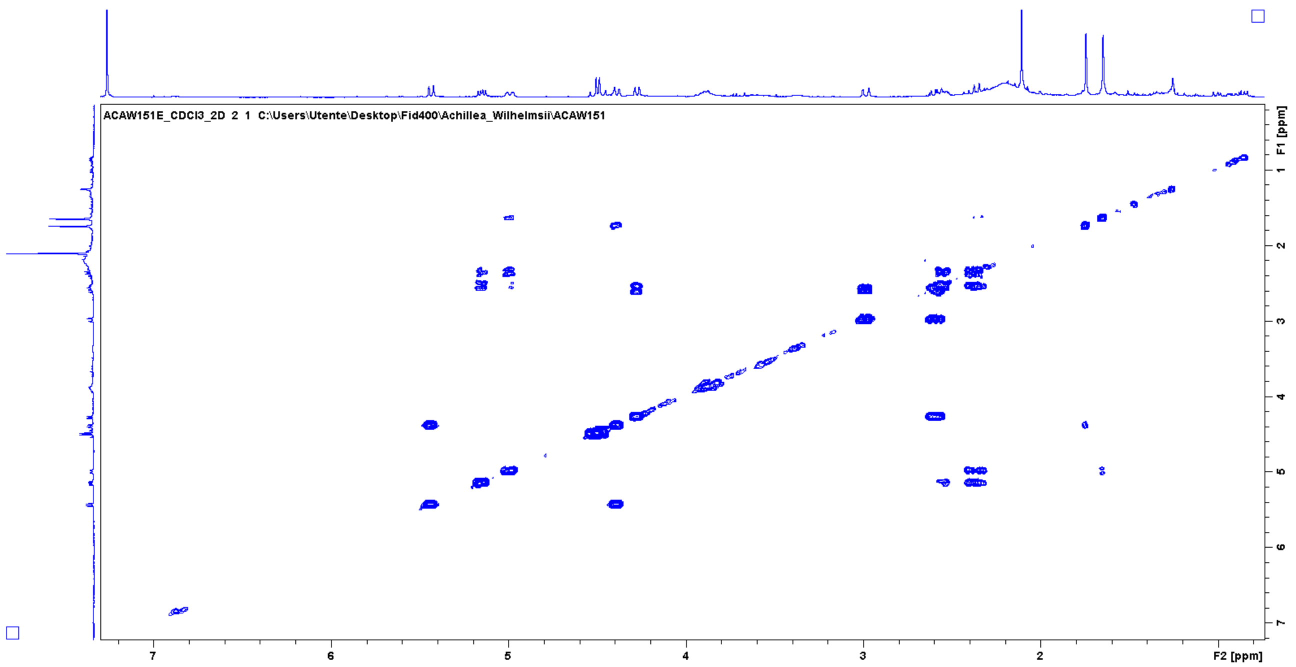

Figure S5. 2D COSY NMR spectrum of wilhelmsolide ($CDCl_3$)

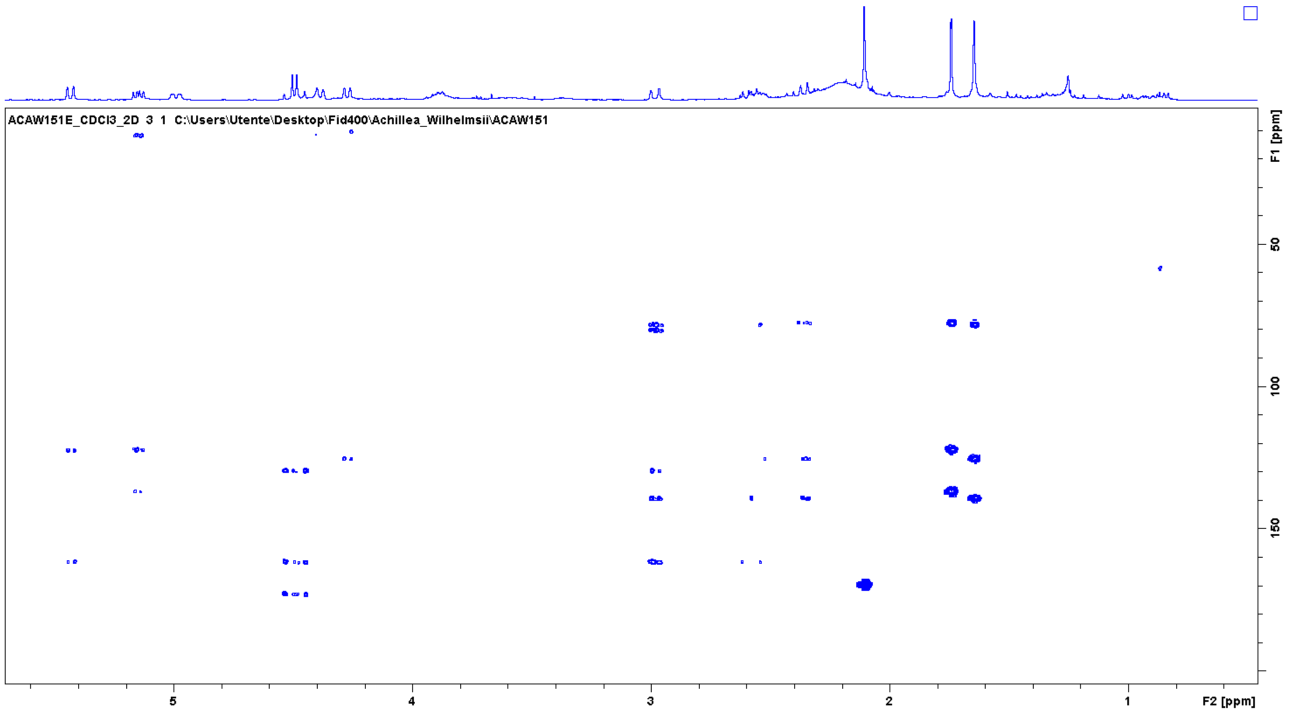

Figure S6. 2D HMBC NMR spectrum of wilhelmsolide ($CDCl_3$)

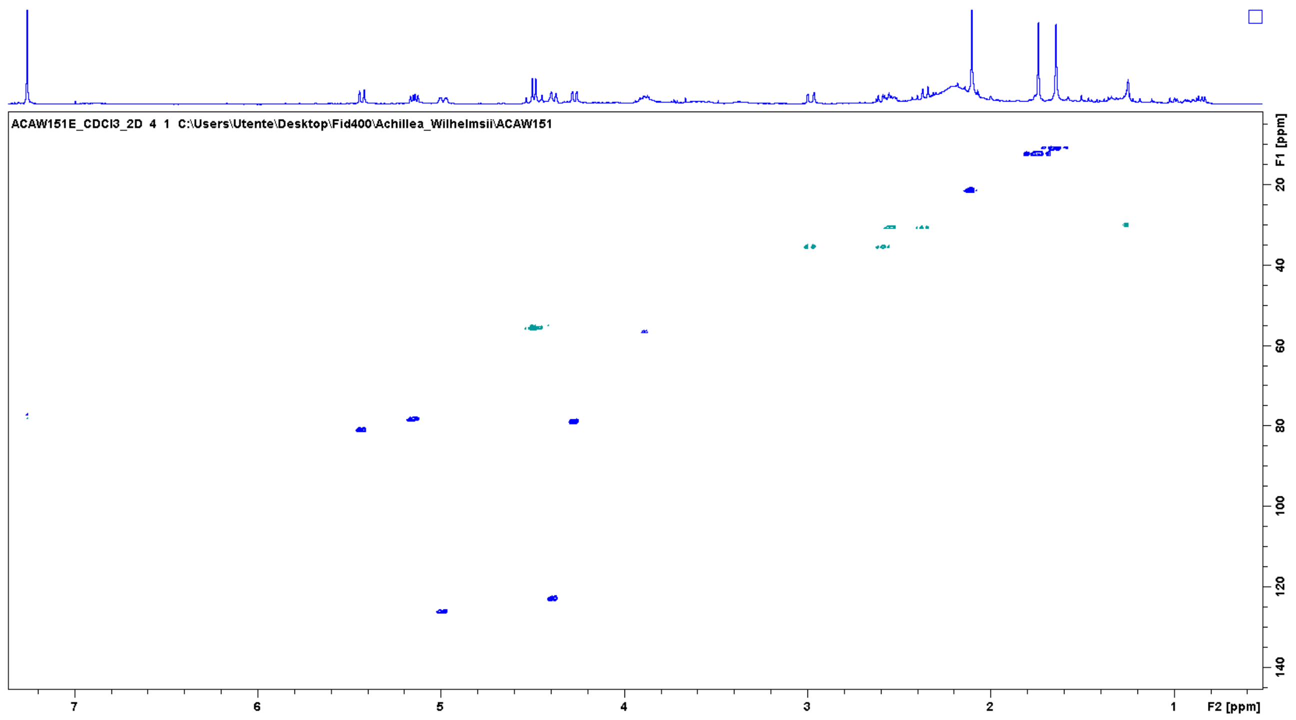

Figure S7. 2D HSQC NMR spectrum of wilhelmsolide ($CDCl_3$)
